# A Novel Technique for the Assessment of Preoperative Cardiovascular Risk: Reactive Hyperemic Response to Short-Term Exercise

**DOI:** 10.1155/2013/837130

**Published:** 2013-04-10

**Authors:** Robert Schier, Jochen Hinkelbein, Hanke Marcus, Ashley Smallwood, Arlene M. Correa, Reza Mehran, Randa El-Zein, Bernhard Riedel

**Affiliations:** ^1^Department of Anaesthesiology and Intensive Care Medicine, University Hospital of Cologne, Kerpener Strasse 62, 50937 Cologne, Germany; ^2^Department of Anesthesiology and Pain Medicine, The University of Texas M. D. Anderson Cancer Center, 1515 Holcombe Boulevard, Houston, TX 77030, USA; ^3^Department of Thoracic Surgery, The University of Texas M. D. Anderson Cancer Center, 1515 Holcombe Boulevard, Houston, TX 77030, USA; ^4^Department of Epidemiology, The University of Texas M. D. Anderson Cancer Center, 1515 Holcombe Boulevard, Houston, TX 77030, USA; ^5^Department of Anaesthesia and Pain Medicine, Peter MacCallum Cancer Centre and The University of Melbourne, Locked Bag 1, A'Beckett Street, Melbourne, VIC 8006, Australia

## Abstract

*Background*. Perioperative vascular function has been widely studied using noninvasive techniques that measure reactive hyperemia as a surrogate marker of vascular function. However, studies are limited to a static setting with patients tested at rest. We hypothesized that exercise would increase reactive hyperemia as measured by digital thermal monitoring (DTM) in association to patients' cardiometabolic risk. *Methods*. Thirty patients (58 ± 9 years) scheduled for noncardiac surgery were studied prospectively. Preoperatively, temperature rebound (TR) following upper arm cuff occlusion was measured before and 10 minutes after exercise. Data are presented as means ± SD. Statistical analysis utilized ANOVA and Fisher's exact test, with *P* values <0.05 regarded as significant. *Results*. Following exercise, TR-derived parameters increased significantly (absolute: 0.53 ± 0.95 versus 0.04 ± 0.42°C, *P=0.04*, and % change: 1.78 ± 3.29 versus 0.14 ± 1.27 %, *P=0.03*). All patients with preoperative cardiac risk factors had a change in TR (after/before exercise, ΔTR) with values falling in the lower two tertiles of the study population (ΔTR <1.1%). *Conclusion*. Exercise increased the reactive hyperemic response to ischemia. This dynamic response was blunted in patients with cardiac risk factors. The usability of this short-term effect for the preoperative assessment of endothelial function warrants further study.

## 1. Introduction

The physiological response of peripheral vasodilation during and shortly after exercise is affected by several factors. The vascular endothelium plays a central role in the regulation of vascular tone via nitric oxide, which has a key role in endothelial function, and is involved in exercise-induced vasodilation [1, 2]. Impaired endothelial function is promoted by injury from mechanical forces and processes related to cardiovascular risk factors including ageing [3], hypertension [4], dyslipidaemia [5], impaired fasting glucose [6, 7], insulin resistance [8], hyperhomocysteinemia [9], smoking [10, 11], or acute postprandial hypertriglyceridemia [12]. With an increasing incidence of these risk factors among the patient population presenting preoperatively, noninvasive assessment of endothelial-dependent vascular function in response to exercise might be a diagnostic tool gaining importance in the preoperative risk assessment.

Despite advances in perioperative care, patients undergoing major noncardiac surgery continue to experience a high incidence of postoperative morbidity (15–36%) and mortality (4.8–10.9%, e.g., following pneumonectomy), with increased health care expenditure [13, 14]. Cardiovascular risk factors predispose to perioperative morbidity and mortality, with evidence that patients with microvascular dysfunction undergoing cardiovascular interventions are at increased risk for postoperative complications [15, 16].

Given that the noncardiovascular surgical population increasingly presents with multiple cardiovascular risk factors [17], there is need to explore the role of endothelial dysfunction in this population from a clinical point of view. Recent studies investigating DTM have shown that impaired vascular reactivity correlated with the extent of myocardial perfusion defect and was found in patients with coronary artery disease, metabolic syndrome, and diabetes mellitus [18, 19]. It is increasingly recognized as a diagnostic tool for cardiovascular risk assessment [20–22].

The noninvasive assessment of reactive hyperemia *in response to exercise*, as a surrogate marker of endothelial-dependent vascular function, has not been described in the literature at this point. Therefore, we tested the hypothesis that acute exercise would increase reactive hyperemia regardless of changes in blood pressure, heart rate, and body temperature (primary endpoint). In addition, we tested the hypothesis that a lack of reactive hyperemia increase after exercise would correlate with preoperative cardiovascular risk factors (secondary endpoint).

## 2. Methods

### 2.1. Subjects

Following IRB approval (The University of Texas M.D. Anderson Cancer Center, study protocol no. 2003-0434), thirty consecutive patients scheduled for major noncardiac surgery (esophagectomy or major lung surgery, e.g., lobectomy or pneumonectomy) were prospectively enrolled into this observational trial. Exclusion criteria were any condition that deemed a patient unsatisfactory for surgery after the preanesthetic evaluation. Patients were evaluated with standard preoperative risk scores, including the American Society of Anesthesiologists (ASA) Physical Status Classification System and modified Lee Cardiac Risk Index [23, 24]. 

### 2.2. Study Endpoints

The primary endpoint of this pilot study investigated whether acute exercise would increase reactive hyperemia, a surrogate marker of vascular function, and that this effect would be blunted in the presence of preoperative cardiovascular risk factors (i.e., coronary artery disease, hypertension, diabetes, and obesity).

### 2.3. Measurement of Reactive Hyperemia

**Figure 1:**
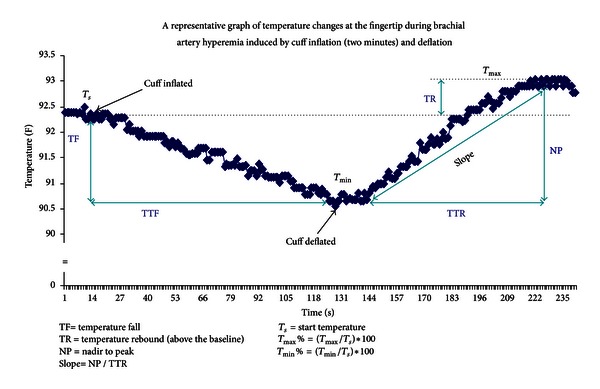
Representative example of a temperature-time trace in response to occlusion-hyperemia.

To ensure consistency, all measurements of reactive hyperemia were performed within one week of scheduled surgery. Measurements were performed before and 10 minutes after exercise in a quiet dimmed room at a controlled ambient temperature (20–25°C) using a VENDYS 5000BC Digital Thermal Monitoring (DTM) system (Endothelix, Inc., Houston, TX, USA). This FDA approved device consists of a computer-based thermometry system (0.006°C thermal resolution), with two special thermocouple fingertip probes designed to minimize the area of skin-probe contact and fingertip pressure. A standard sphygmomanometer cuff and a compressor unit to control cuff inflation and deflation is included to facilitate the occlusion-hyperemia protocol. The test is conducted with the patient at rest for 30 minutes in the supine position, in a quiet, dimmed room with ambient temperature of 24°C to 26°C. VENDYS DTM probes are affixed to the index finger of each hand and after a period of stabilization of basal skin temperature (defined as stabilization within a 0.05°C threshold) the temperature is measured in the index fingers of both hands (of which the right arm only is subjected to occlusion-hyperemia) with an automated, operator-independent protocol. The right upper arm cuff is rapidly inflated to ≥50 mmHg above systolic pressure for 2 minutes and then rapidly deflated to invoke reactive hyperemia distally. Thermal tracings are measured continuously and digitized automatically using a computer-based thermometry system with 0.006°C thermal resolution. Dual channel temperature data is simultaneously acquired at a 1 Hz sample rate.  Figure 1 shows a representative example of a temperature-time trace and the primary DTM-derived measures, related to thermal debt and recovery that were recorded and calculated.

## 3. Cardiopulmonary Exercise Testing (CPET)

Prior to exercise, baseline vitals (heart rate, blood pressure, pulse oximetry, and ECG) and static pulmonary function tests (forced expiratory volume at 1 second, forced vital capacity, and maximal voluntary ventilation) were recorded for all patients. CPET was then performed as a multistage incremental “ramp workload” study using a cycle ergometer and a metabolic cart with standardized exercise software (Medgraphic Cardio-2CP system, Medical Graphics Corporation, St. Paul, MN, USA) for breath-by-breath analysis of gas exchange.

An initial acclimation period consisted of breath-by-breath gas exchange analysis performed in the supine, resting position for five minutes. After acclimation the patient pedaled at 60 rpm with minimal resistance for three minutes (unloaded work). After three minutes, loaded work (increasing pedal resistance, watts per minute) followed a standardized ramp protocol to maximal symptom limited exertion that typically lasted 9–12 minutes. Exercise was terminated by the study patient or by the study investigator if symptoms of cardiovascular, pulmonary distress, and/or fatigue were observed. To ensure consistency, exercise above the anerobic threshold was required for inclusion into the study. Anerobic threshold (AT, mL/kg/min) was defined as the VO_2_ at the inflection point as determined by the modified V-slope method of plotting carbon dioxide excretion (VCO_2_) against oxygen uptake (VO_2_) during increasing exercise intensity, as described by Wasserman et al. [25]. Gas exchange analysis recorded oxygen consumption (VO_2_, mL/kg/min) and carbon dioxide production (VCO_2_, mL/kg/min) at all phases of exercise.

### 3.1. Statistical Analysis

The study sample size determination was based on data from a previous study by Harris et al. who enrolled nine patients to detect an increase of reactive hyperemia, as measured by flow-mediated dilation of the brachial artery, immediately after 45 min of exercise on a treadmill at 50% of their VO_2_ peak. We calculated that a sample of thirty patients would need to be enrolled to achieve 80% power to detect a log-linear trend in the primary endpoint assuming that the percentage increase of reactive hyperemia after exercise, as measured by TR, was 50 percentage points. Descriptive statistics were used to summarize the patients' demographic, clinical, and TR measures. The relative changes from baseline (before exercise) and after-exercise (10 minutes after peak exercise) were analyzed using repeated measures (ANOVA) and Wilcoxon signed ranks test.

Fisher's exact test was used to analyze for an association of perioperative variables—including patients' comorbidities (i.e., obesity, abdominal obesity, coronary artery disease, and Modified Lee Cardiac Risk Index) with TR measures when tertiles were used as cutoff points. A *P* value of less than 0.05 was considered to indicate statistical significance. Statistical analyses were carried out using SAS 9.1 (SAS Institute, Cary, NC, USA) and S-Plus (version 8; Insightful Corp., Seattle, WA, USA).

## 4. Results

### 4.1. Clinical and Demographic Characteristics of the Study Participants

Thirty patients (18 males and 11 females) with mean age of 58 ± 10 years scheduled for major noncardiac surgery were enrolled in the study. Twenty-eight (93%) patients had an increased perioperative risk with an ASA score >2; thirteen (46%) patients had cardiovascular risk factors, for example, hypertension and dyslipidemia; and twenty-one (70%) patients were current smokers.  Table 1 summarizes the demographic and clinical characteristics of the study population.

### 4.2. Reactive Hyperemia (TR) before and after Exercise

Table 2 summarizes the vital signs (heart rate and blood pressure) and the reactive hyperemia measures before and after exercise. The heart rate was significantly increased 10 min after exercise when compared to baseline (mean: 75 ± 10.58 versus 76 ± 19.88 min^−1^; *P* = 0.021). There were no differences in blood pressure before and after exercise. The starting temperature at the beginning of the reactive hyperemia measurement did not differ before and after exercise (mean: 32.84 ± 1.78 versus 32.23 ± 2.01°C; *P* = 0.147).

**Figure 2:**
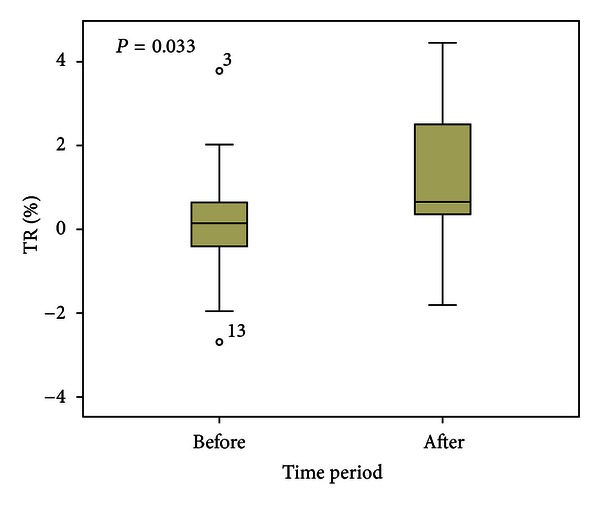
Increase of reactive hyperemia (temperature rebound, TR%) 10 minutes after peak exercise.

Reactive hyperemia was significantly increased 10 min after exercise with an absolute TR increase of 0.04 ± 0.42 versus 0.53 ± 0.95°C, *P* = 0.035 and a relative TR increase of 0.14 ± 1.27 versus 1.78 ± 3.29%, *P* = 0.033 (Figure 2). Area under the curve (AUC) of the TR slope was significantly lower after exercise with AUC 15 sec: 14.89 ± 4.70 versus 11.92 ± 5.26, *P* = 0.019; AUC 30 sec: 29.01 ± 9.04 versus 23.29 ± 10.23, *P* = 0.017; AUC 45 sec: 41.50 ± 12.86 versus 33.34 ± 14.53, *P* = 0.017; and AUC 60 sec: 52.11 ± 16.15 versus 41.85 ± 18.12, *P* = 0.020.

There was no association between clinical characteristics and low TR values (2 lower tertiles) when compared to high TR values (upper tertile) (Table 3). 

## 5. Discussion

The principal finding of the present study is that a single episode of acute exercise above the anerobic threshold enhanced the reactive hyperemia, a surrogate marker of endothelial function. These results imply that a short period of exercise enhances cutaneous perfusion and is associated with an increase in the release and/or bioactivity of endogenous vasodilatative mediators (e.g., NO) in the endothelial cells of skin vasculature. We were able to measure this short-term effect with the use of digital thermal monitoring (DTM) of temperature rebound (TR), which provides a noninvasive assessment of vascular function.

However, the diagnostic value of our findings in terms of preoperative assessment of endothelial dysfunction warrants further research. Although we found that patients with preoperative cardiac risk factors and postoperative complications were within the lower 2 tertiles of the study population (ΔTR < 1.1%), this observation needs to be validated in a larger patient population.

In agreement with our findings, previous work has demonstrated that acute exercise increases skin blood flow and cutaneous vascular conductance accompanied by enhanced plasma NO metabolite levels and acetylcholine-induced cutaneous perfusion [26]. These authors suggested that endothelium-dependent dilation in skin vasculature is enhanced by moderate exercise training and reversed to the pretraining state with detraining.

Furthermore, our observations suggest that this effect can be reproduced by a single episode of exercise above the anerobic threshold increasing the aerobic capacity and vascular responsiveness to acute exercise. 

In contrast, a previous study investigating on the effect of 6 months of aerobic exercise in patients with type 2 diabetes mellitus was not able to show an improvement of microvascular dysfunction [27]. The authors interpreted their negative results with the hypothesis that micro- and macrocirculation respond differently to the exercise stimulus. We were able to observe a significant increase of reactive hyperemia after a short exercise stimulus. However, it remains unclear how long this effect would have lasted on and we suggest that our observed physiological response to exercise has rather diagnostic than therapeutic value.

Our study has implications for preoperative assessment of endothelial function, as the observed increased reactive hyperemic signal shortly after exercise may serve as a diagnostic tool. Impairment of endothelial function is a precursor for cardiovascular disease and precedes the morphological changes associated with atherosclerosis in the blood vessels [28] and the clinical manifestations of its associated complications (e.g., myocardial infarction, stroke) [29, 30]. Furthermore, any transient inflammatory burden or a systemic inflammatory state also adversely affects endothelium-dependent vascular function with consequent increase in risk for cardiovascular complications [31, 32]. In the perioperative context, inflammatory mediator release associated with surgical trauma has been shown to impair vascular function and correlate with both the duration and extent of major surgery [31–35]. This effect may be additive to the underlying endothelial dysfunction that is inherent in certain surgical patients as a result of their preoperative comorbidity burden and thus plays a significant role in certain perioperative complications (e.g., perioperative myocardial infarction, poor wound healing, ALI, and sepsis) [33, 35]. 

## 6. Conclusions

Based on our results, we suggest that the preoperative assessment of endothelial function using reactive hyperemia in response to exercise gains clinical importance as a potential risk assessment tool in the prevention of perioperative complications and should be further studied in a larger patient population.

## Figures and Tables

**Table 1:** Clinical characteristics.

	*n*	Mean (±SD)	Median (range)
Age, y	30	58 (±9.93)	59 (45–70)
Height, m	30	1.70 (±0.11)	1.70
Weight, kg	30	83 (±18.62)	81
Waist, cm	28	104 (±38.48)	98
BMI, (kg/m)^2^	30	28 (±4.76)	28
PreOp hemoglobin, mg/dL	30	13 (±1.16)	13
PreOp fasting glucose, mg/dL	30	103 (±24.63)	100
PreOp creatinine, mg/dL	28	1.03 (±0.24)	1.00
Length of hospital stay, d	30	12 (±13.91)	7
Length of ICU stay, d	30	4 (±13.39)	0

	*n* (%)		

Sex, female	11 (37)		
Obesity, *n* (%)	10 (33)		
Abdominal obesity, *n* (%)	13 (46)		
Smoker, *n* (%)	21 (70)		
Coronary artery disease**, *n* (%)	1 (3)		
Hypertension, *n* (%)	13 (43)		
Diabetes, *n* (%)	4 (13)		
Dyslipidemia, *n* (%)	13 (43)		
Statin therapy, *n* (%)	5 (17)		
*β*-Blocker therapy, *n* (%)	6 (20)		
ACE-inhibitor therapy, *n* (%)	4 (13)		
ASA risk score > 2, *n* (%)	28 (93)		
Lee Cardiac Risk Index > 2, *n* (%)	3 (10)		
Chemotherapy, *n* (%)	13 (43)		
Radiation therapy, *n* (%)	10 (33)		

**Patient status after myocardial infarction (with or without intervention).

**Table 2:** Reactive hyperemia (TR) before and after exercise.

	Before exercise			After exercise (10 min after)			*P* values*
	*N*	Mean	Std deviation	*N*	Mean	Std deviation	
Starting temperature (°C)	30	32.84	1.78	30	32.23	2.01	0.147
Temperature rebound (TR°C)	30	0.04	0.42	30	0.53	0.95	0.035*
Temperature rebound (TR%)	30	0.14	1.27	30	1.78	3.29	0.033*
Area under curve after 15 sec	30	14.89	4.70	30	11.92	5.26	0.019*
Area under curve after 30 sec	30	29.01	9.04	30	23.29	10.23	0.017*
Area under curve after 45 sec	30	41.50	12.86	30	33.34	14.53	0.017*
Area under curve after 60 sec	30	52.11	16.15	30	41.85	18.12	0.020*
Heart rate (bpm)	27	75	10.58	28	76	19.88	**0.021 **
Systolic blood pressure (mmHg)	27	128	16.94	28	132	16.35	0.216
Diastolic blood pressure (mmHg)	27	76	6.09	28	79	9.06	0.081
Mean blood pressure (mmHg)	27	94	11.59	28	98	2.01	0.094

*Wilcoxon signed ranks test.

**Table 3:** Clinical characteristics and tertiles of TR % change after exercise (pre-/postexercise difference).

	*n*	Lower 2 tertiles (<−0.0952 and <1.1162)	Upper tertiles (≥1.1162)	*P* value*
Age, y	30	57.5 ± 11.3	59.1 ± 6.7	0.685
Sex, *n* (%) female	11	6 (55)	5 (45)	0.425
Height, m	30	1.7 ± 0.1	1.7 ± 0.1	0.589
Weight, kg	30	84.5 ± 19.3	79.5 ± 17.8	0.501
Waist, cm	28	107.5 ± 46.2	95.9 ± 10.1	0.465
BMI, (kg/m)^2^	30	28.8 ± 5.4	27.7 ± 3.3	0.567
Obesity, *n* (%)	10	8 (80)	2 (20)	0.419
Abdominal obesity, *n* (%)	13	8 (62)	5 (38)	0.505
Smoker, *n* (%)	21	12 (57)	9 (43)	0.204
Coronary artery disease**, *n* (%)	1	1 (100)	0 (0)	1.000
Hypertension, *n* (%)	13	11 (85)	2 (15)	0.119
Diabetes, *n* (%)	4	4 (100)	0 (0)	0.272
Dyslipidemia, *n* (%)	13	8 (62)	5 (38)	0.705
Statin therapy, *n* (%)	5	3 (60)	2 (40)	1.000
*β*-Blocker therapy, *n* (%)	6	6 (100)	0 (0)	0.074
Aspirin therapy, *n* (%)	5	5 (100)	0 (0)	0.140
ACE-inhibitor therapy, *n* (%)	4	3 (75)	1 (25)	1.000
ASA risk score > 2, *n* (%)	28	19 (68)	9 (32)	0.615
Lee Cardiac Risk Index > 2, *n* (%)	3	3 (100)	0 (0)	0.107
Chemotherapy, *n* (%)	13	7 (54)	6 (46)	0.255
Radiation therapy, *n* (%)	10	7 (70)	3 (30)	0.101
PreOp echo/EF, %	17	61.4 ± 3.8	62.4 ± 6.9	0.709
PreOp hemoglobin, mg/dL	30	13.3 ± 1.0	13.5 ± 1.4	0.705
PreOp fasting glucose, mg/dL	30	106.7 ± 28.6	95.8 ± 12.0	0.260
PreOp creatinine, mg/dL	28	1.0 ± 0.3	1.0 ± 0.2	0.509
Length of hospital stay, d	30	11.2 ± 11.7	12.6 ± 18.32	0.800
Length of ICU Stay, d	30	2.4 ± 9.0	6.3 ± 19.9	0.462

*Fisher's exact test.

**Patient status after myocardial infarction (with or without intervention).
